# Simultaneous Effects of Thermal Cycling and Shear on Flow Instabilities of Phase-Change Nanoemulsions Measured by Rheo-NMR and MRI Velocimetry

**DOI:** 10.1021/acs.jpclett.5c00307

**Published:** 2025-03-24

**Authors:** Jungeun Park, Benjamin Kohn, Robert J. Messinger, Ulrich Scheler

**Affiliations:** † Department of Chemical Engineering, The City College of New York, CUNY, New York, New York 10031 United States; ‡ Department for Multi-Scale Characterization, Leibniz-Institut für Polymerforschung Dresden e.V., Hohe Str. 6, 01069, Dresden, Germany

## Abstract

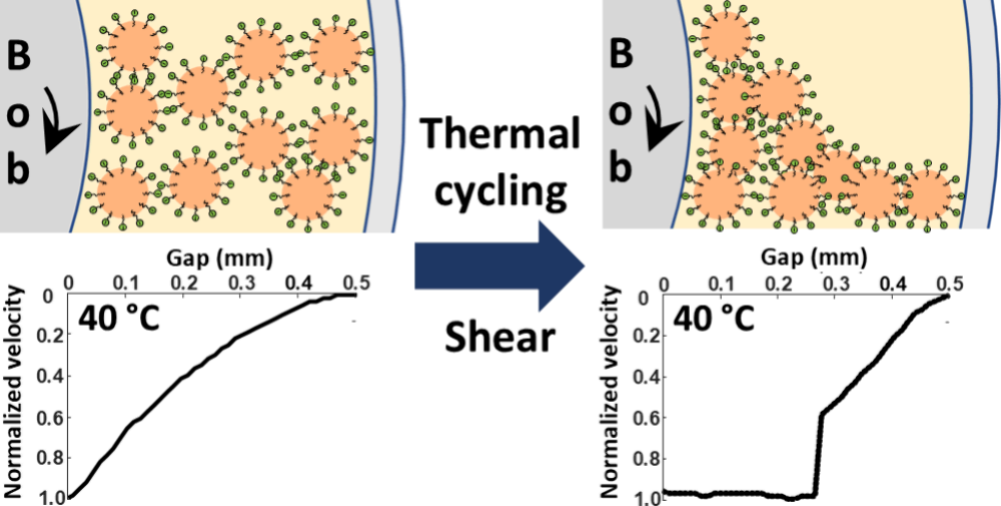

Organic phase change materials (PCMs) for thermal energy storage can be emulsified in water in the presence of surfactants to enable their use as pumpable heat transfer fluids. However, PCM nanoemulsions often exhibit instabilities during thermal cycling and shear flow that limit their use. To investigate their combined effects, rheological nuclear magnetic resonance (rheo-NMR) spectroscopy and magnetic resonance imaging (MRI) velocimetry methods were applied on a model octadecane-water-stearic acid system. Rheology measurements indicated that the viscosity exhibited hysteresis during thermal cycling, which correlated with the solid fraction of octadecane. Fluid velocity profiles and concentration distributions of liquid octadecane were noninvasively measured in a Searle cell. Nonlinear fluid velocity profiles developed after the octadecane solid-to-liquid phase transition, which recovered to linear profiles after octadecane melting at lower shear rates but notably not at higher shear rates. Nonuniform concentrations of liquid octadecane were measured during thermal cycling, a result of shear-induced mass transport, which causes local viscosity gradients that can lead to hydrodynamic instabilities and nonlinear fluid velocity profiles. The results not only show how shear affects flow instabilities in PCM nanoemulsions during thermal cycling but also demonstrate that this NMR methodology is a powerful tool for noninvasively measuring flow and concentration profiles in complex fluids.

Thermal energy storage systems store energy by either heating or cooling a storage medium, such as molten salts, water, or phase change materials (PCMs).^1−4^ This stored energy can be redistributed for various thermal applications including solar energy storage, building climate control, or industrial processes.^5^ PCMs undergo thermodynamic phase transitions (e.g., solid to liquid, or vice versa) within a specific temperature range, enabling energy to be stored and released in the latent heat of the thermodynamic phase change.^3^ Among PCMs, organic PCMs, such as paraffins and fatty acids, offer several key benefits that make them desirable for thermal energy storage and management.^3,6^ They have greater energy storage densities compared to inorganic PCMs and are available with a wide range of melting points, enabling design and control of specific temperature conditions.^3,6^ In addition, they are safe, cost-effective, and noncorrosive. However, their lower thermal conductivity is a major issue that can slow rates of heat transfer, limiting applications where rapid heating or cooling is required. Another critical issue is supercooling, which occurs when a PCM is cooled below its melting temperature but remains in a metastable liquid state.^1^ Supercooling can bring about unpredictable phase change behavior, lead to delayed heat release, and reduce the thermodynamic energy efficiency of the system.

Organic PCM nanoemulsions, composed of oil droplets (10–500 nm)^7,8^ stabilized by surfactants in a continuous aqueous phase, enhance heat transfer between PCM and surroundings due to increased surface area, significantly improving heat exchange rates.^4,9,10^ They can be used as heat transfer fluids, as they are pumpable above the melting temperature of the continuous phase (e.g., water) and exhibit reduced viscosities, even when the dispersed PCM phase is in the solid state. Surfactants lower the interfacial tension by adsorbing at the interface between immiscible liquids, creating electrostatic and steric barriers that prevent droplet coalescence during emulsification.^11^ Nanoemulsions are kinetically stable but thermodynamically unstable, as the droplets tend to coalesce to reduce their interfacial energy.^7,9,11^ Therefore, nanoemulsion droplets spontaneously coalesce over time and eventually separate into individual phases.

Instability issues such as droplet coalescence, creaming or sedimentation, and Ostwald ripening affect the long-term stability of PCM nanoemulsions, limiting their practical applications in heat transfer systems.^4,10^ In particular, the instability of PCM nanoemulsions can be accelerated by repeated melting and freezing processes, which can be further exacerbated by shear flow during use.^1,12^ For example, in an oil-in-water PCM emulsion composed of 10 vol % hexadecane, a substantial reduction in pressure drop and the local convective heat transfer coefficient were observed at the PCM melting point within a flow loop.^13^ In a 10 wt % beeswax emulsion, large amplitude oscillations in the flow rate and surface temperatures showing unusual heat transfer instabilities have been reported at the phase change temperature in a circular pipe.^14^ The viscosity of a 35 wt % octadecane emulsion increased from 50 to 85 mPa·s over 100 thermal-mechanical cycles in a flow loop.^15^ Typically, the apparent viscosity of PCM nanoemulsions decreases as the temperature increases, and the mass fraction of PCM decreases. However, the apparent viscosity of PCM nanoemulsions has shown significant variation, potentially due to the different particle sizes involved.^16^ Pumping the PCM nanoemulsions during thermal cycling generates mechanical and thermal stresses on the PCM nanoemulsions. These stresses can lead to phase instability, causing an increase in pressure drop due to an increase in viscosity or a variation in the heat transfer rate during the PCM phase change, which can significantly impact flow characteristics and the pump work.

To achieve long-term flow and heat transfer stability of PCM nanoemulsions, it is crucial to understand how the flow field and oil droplet concentrations evolve upon both thermal cycling and shear. However, such quantities are challenging to measure, while underlying hydrodynamic problems associated with phase instability are poorly understood. In a previous study,^17^ we studied how the molecular-level environments and dynamics of the surfactants and oil phase changes in this model PCM nanoemulsion change upon thermal cycling by liquid-state NMR spectroscopy, explaining, in part, the molecular origins of phase instability upon thermal cycling. To understand the combined effects of thermal cycling and shear on PCMs nanoemulsions, it is essential to investigate noninvasively the flow dynamics and rheological properties of PCM nanoemulsion under variable temperature and shear.

These measurements can be achieved noninvasively by using rheological nuclear magnetic resonance (rheo-NMR) spectroscopy in combination with magnetic resonance imaging (MRI) velocimetry. Rheo-NMR is a powerful technique for the noninvasive measurements of molecular-level structures and dynamics under shear. It has been applied in various fields, including material science, polymer chemistry, pharmaceuticals, and food industries.^18,19^ Complex fluids such as emulsions, suspensions, polymers, and micellar solutions have both solidlike and liquidlike characteristics; thus, when subjected to large deformational flows, their physical properties are generally nonlinear, often anisotropic, and spatially heterogeneous. By introducing magnetic field gradients while under shear, rheo-NMR enables acquisition of spatially resolved NMR spectra under flow, providing information on how the molecular-level environments and dynamics change under applied mechanical forces.^20^ In addition, different individual components within a complex fluid can be distinguished and analyzed by measuring their NMR chemical shifts and relaxation times (e.g., longitudinal *T*_1_ and transverse *T*_2_ times). The NMR chemical shift is a diamagnetic shielding effect that depends on the local electronic environment and can be used to identify different chemical species or functional groups. Meanwhile, relaxation times generally reflect molecular motions. As NMR spectroscopy is a quantitative method, concentration distributions of specific components can also be measured. On the other hand, MRI velocimetry enables the flow field to be spatially resolved, revealing flow patterns and velocity gradients within the fluid noninvasively and without any requirement of optical transparency. MRI velocimetry uses a pulsed-gradient spin–echo (PGSE) NMR experiment with additional flow encoding by magnetic field gradients, combined with other MRI techniques.^21^

Both rheo-NMR and MRI velocimetry thus provide nondestructive and complementary information about complex fluids, enabling researchers to achieve a comprehensive picture of their molecular scale and macroscopic behavior under shear.^19,22^ For example, rheo-NMR and MRI velocimetry analyses in wormlike micelle systems revealed shear-induced changes (e.g., shear banding) in the velocity profiles and a transition from a nematic phase in the high stress region to isotropic phase in the low stress region.^23^ Rheo-NMR was used to study polymer chain dynamics in a Couette cell showed that chain entanglement restricts motion, resulting in less averaging of dipolar coupling and consequently shorter transverse (*T*_2_) relaxation times.^24^

Here, to better understand the simultaneous effects of thermal cycling and shear on PCM nanoemulsions, fluid velocities, and concentration distributions of a model PCM nanoemulsion system were measured using ^1^H rheo-NMR and MRI velocimetry. The model PCM nanoemulsion system consisted of octadecane as the dispersed organic PCM phase, dilute aqueous NaOH as the continuous phase, and stearic acid as the surfactant. Rheology and dynamic light scattering (DLS) measurements were performed to measure how the viscosity changed upon temperature and emulsion droplet sizes change under shear and thermal cycling. Fluid velocity profiles and concentration distributions of liquid octadecane were noninvasively measured in a Searle cell during thermal cycling at different shear rates.

A model PCM nanoemulsion containing 20 wt % octadecane as an oil phase, 2.5 wt % stearic acid as a surfactant, and 77.5 wt % aqueous 0.05 M NaOH as a medium were designed and investigated in a recent study from our group.^17^ To increase shear effects and signal-to-noise ratios in the rheo-NMR experiment, the mass fraction of octadecane increased to 30 wt %, while the surfactant-to-oil mass ratio was fixed. Thus, PCM nanoemulsions containing 30 wt % octadecane, 3.75 wt % stearic acid, and 66.25 wt % aqueous 0.05 M NaOH for all rheo-NMR and MRI velocimetry measurements.

To understand how thermal cycling and shear affect flow instabilities in PCM nanoemulsions, the relative fractions of liquid and solid oils must first be characterized as a function of temperature. The liquid fraction of octadecane within the oil phase, *f*, was obtained from liquid-state ^1^H single-pulse NMR measurements, where the integrated ^1^H signal intensity of the octadecane alkyl groups represents the liquid content. Note that only liquid octadecane is observed, as fast, isotropic molecular motions average away anisotropic NMR interactions (in particular, magnetic dipole–dipole interactions and chemical shift anisotropy) that would otherwise broaden the ^1^H signals of solid octadecane below the noise. The alkyl signals of the surfactant were not distinguished from the octadecane but do not play a separate role. Samples were thermally cycled from 40 to 5 °C, then back to 40 °C, to change the phase of octadecane from liquid to solid while maintaining water in its liquid state. Liquid-state ^1^H single-pulse NMR experiments were acquired on PCM nanoemulsions containing 30 wt % octadecane at different temperatures, including 40, 26, and 17 °C upon cooling and 26, 27, and 40 °C upon heating. The liquid content of octadecane in PCM nanoemulsions with 30 wt % octadecane was in excellent quantitative agreement with those obtained in our previous study,^17^ which used a 20 wt % octadecane model PCM nanoemulsion (Figure 1). The results show that the degree of supercooling is independent of the octadecane content and shear within these composition and flow regimes. For a comparison to the viscosity of the nanoemulsion, the data have been reversed to plot the solid fraction in the oil phase under the assumption that at high temperature the oil is completely molten.

**Figure 1:**
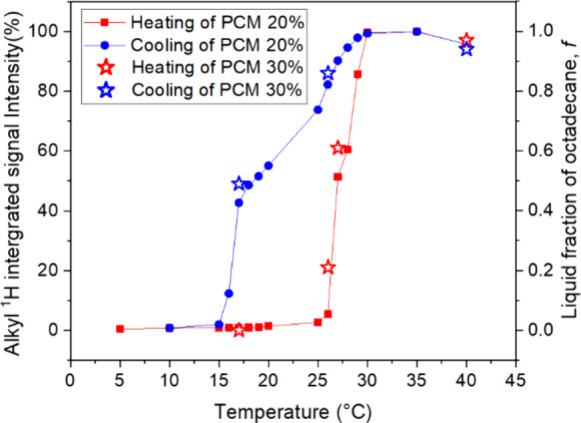
Total alkyl ^1^H NMR integrated signal intensity, and corresponding liquid fraction of octadecane (*f*), in PCM nanoemulsions containing 30 wt % octadecane rotated at a frequency of 28 s^–1^ (this work) and PCM nanoemulsions containing 20 wt % octadecane under static conditions. Adapted from ref (17). Copyright 2024 The Authors.

Viscosity is one of the key rheological characteristics that significantly influences the pressure drop and pumping power during the application of PCMs nanoemulsions as heat transfer fluids.^25^ Both the shear rate and temperature have a significant impact on emulsion viscosity. The dynamic viscosity as a function of shear rate was obtained at 25 °C (Figure S1). The dynamic viscosity decreases with increasing shear rate, exhibiting non-Newtonian shear-thinning behavior.^26^ The viscosity of this model PCM nanoemulsion tends to remain at 0.02 Pa·s above a shear rate of 630 s^–1^. Shear-thinning rheology has been observed in worm-like micellar solution due to changes in their local alignment and interactions, where microstructural rearrangements reduce resistance to flow (i.e., viscosity) and result in shear-thinning behavior.^27^ Micellar systems also exhibit rheological behavior with similar origins, where alignment^28^ or microstructure^29^ changes occur as shear increases, reducing viscosity and causing shear thinning behavior.

The viscosities of the PCM nanoemulsion were also measured using fixed shear rates at different temperatures (Figure 2a). Samples were heated to 40 °C before the experiment so that they followed the same thermal path. The mean droplet sizes before and after the fixed-shear viscosity tests were measured by DLS (Figure 2b). Generally, emulsions having smaller droplets exhibit higher viscosities and stronger shear thinning effect.^30^ Viscosities measured at 40 °C were significantly lower than those measured at 25 °C at both the 500 and 1400 s^–1^ shear rates. The phase transition of octadecane from solid to liquid thus reduces the viscosity of the PCM nanoemulsion. Solid particles undergo limited deformation when subjected to shear stress compared to liquid droplets, resulting in higher viscosity compared to the liquid droplets.^26^ Viscosity measured at 40 °C was greater at a shear rate of 1400 s^–1^ (4.40 mPa·s), compared to 500 s^–1^ (3.63 mPa·s), as expected due to the shear thinning behavior and was relatively constant over the test period of 3500 s. The mean droplet sizes of samples measured at 40 °C changed from 200 to 196 nm after the viscosity measurement at 500 s^–1^ and from 192 to 193 nm at 1400 s^–1^, respectively. Interestingly, at 25 °C, the viscosity was initially greater at 1400 s^–1^ compared to 500 s^–1^, though the viscosity at 25 °C decreased with time. This decrease in viscosity may be associated with phase coalescence of nanoemulsions droplets. The mean droplet sizes measured at 25 °C increased from 202 to 261 nm after the viscosity measurement at 500 s^–1^ and from 196 to 234 nm at 1400 s^–1^, respectively. Notably, the mean droplet sizes increased significantly after applying shear at 25 °C, indicating that the effect of shear is greater below the melting temperature of octadecane.

**Figure 2:**
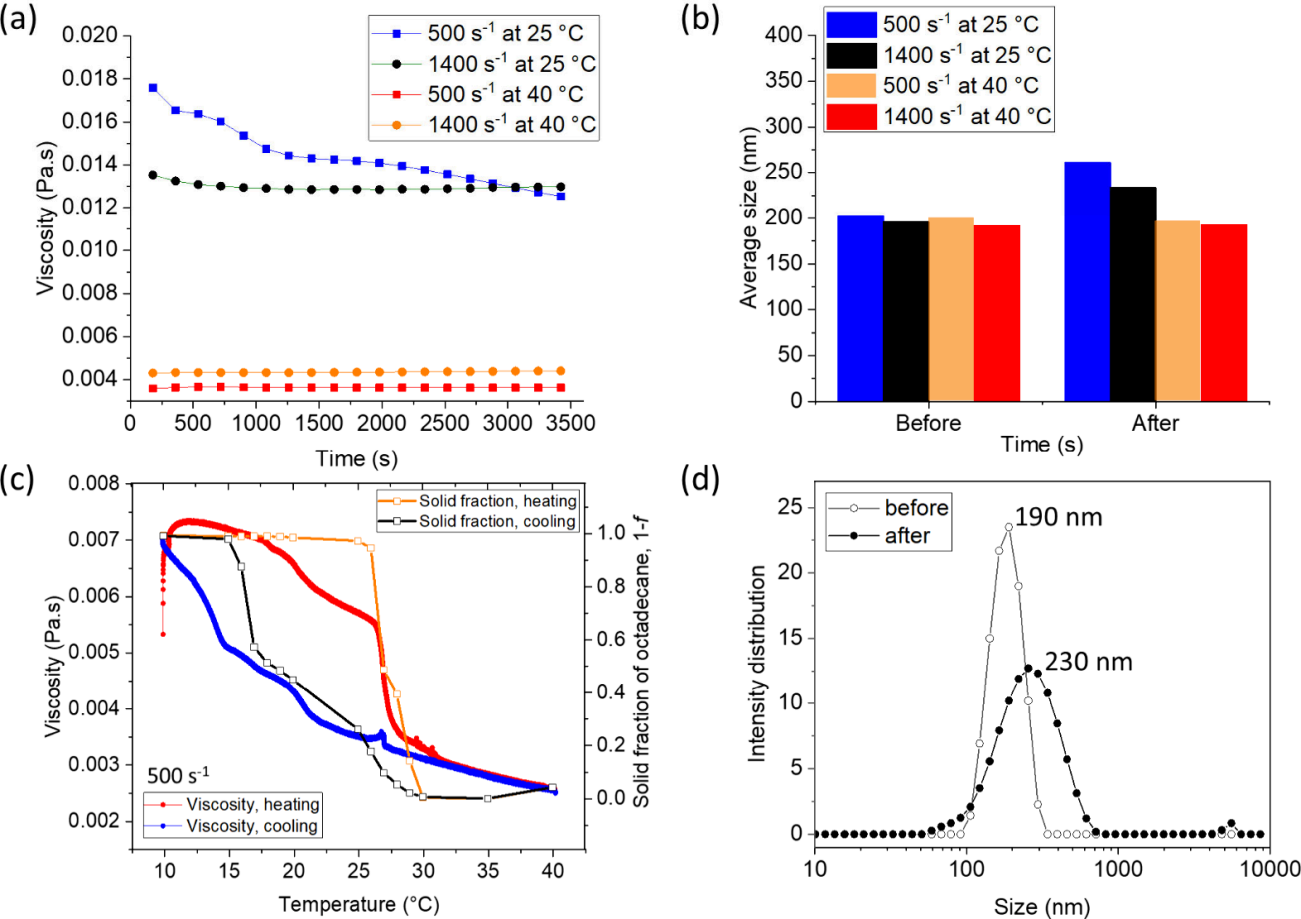
(a) Viscosities of PCM nanoemulsions (20 wt % octadecane, 2.5 wt % stearic acid, 77.5 wt % aqueous 0.05 M NaOH) measured using a rheometer with a 500 or 1400 s^–1^ shear rate and at 25 or 40 °C. (b) Mean droplet sizes before and after the viscosity measurement. (c) Variable-temperature viscosity measurement conducted at a constant shear rate of 500 s^–1^. The solid fraction of octadecane (1-*f*) determined by liquid-state ^1^H single-pulse NMR measurement is also shown for comparison. (d) Droplet size and distribution determined by DLS before and after the variable-temperature viscosity measurement in (c).

To investigate the viscosity of the PCM nanoemulsions during the octadecane phase change, a variable-temperature viscosity measurement was conducted at a constant shear rate of 500 s^–1^ over one thermal cycle. During the viscosity measurement, the sample was heated from 10 to 40 °C and then cooled from 40 to 10 °C at a shear rate of 500 s^–1^ (Figure 2c). The viscosity changes during thermal cycling and shear were correlated to the solid fraction of octadecane, 1-*f*. Initially, the viscosity increases upon heating at 10 °C due to yield stress. Subsequent comparison of the viscosity and solid fraction of octadecane during thermal cycling reveals that they are strongly correlated. The viscosity of the PCM nanoemulsion decreases while heating as the solid fraction of octadecane concomitantly decreases. Similarly, the viscosity increases while cooling as the solid fraction of octadecane increases. Deviations between changes in viscosity and solid fraction of octadecane may result from incomplete melting or freezing during viscosity measurements, conducted at a rate of 0.5 °C/min. In contrast, liquid-state ^1^H single-pulse NMR measurements more accurately capture the equilibrium or metastable thermodynamic state of octadecane. Thus, the observed hysteresis of viscosity is due to the solid fraction of octadecane and is significantly affected by its supercooling.

The mean droplet size was measured before and after the variable-temperature viscosity measurements (Figure 2d). After one complete thermal cycle conducted at a shear rate of 500 s^–1^, the droplet size increased from 190 to 230 nm and the distribution broadened significantly, indicating coalescence and flocculation has occurred, a sign of phase instability. In our previous work,^17^ we showed that the mean droplet size of the PCM nanoemulsion does not change after one complete thermal cycle in the absence of shear. Thus, the results indicate that shear has a significant impact on the PCM nanoemulsion phase stability, amplifying the effects of thermal cycling.

To understand the combined effect of thermal cycling and shear at a local level, we measured the velocity profiles of the PCM nanoemulsion upon thermal cycling and shear *in situ* by using rheo-NMR and MRI velocimetry. The schematic image of the experimental Searle cell setup is shown illustrating the concentric double cylinder (Figure 3a) and a top view of a cross-section of the cell (Figure 3b). Samples were cooled down from 40 to 5 °C, then heated to 40 °C. Velocity profiles were obtained at 40, 26, and 17 °C upon cooling and 26, 27, and 40 °C upon heating. A bob rotation frequency of 0.25 or 0.50 Hz was used, corresponding to shear rates of 14 or 28 s^–1^. The symmetry inherent in the cylindrical setup and the resulting flow pattern allows for a reduction in the necessary data, simplifying the experiments. Using double-slice selection along the center of the bob region where extensional flow occurs, ^1^H velocity profiles were obtained of the PCM nanoemulsion (Figure 3c), primarily comprising the H_2_O signal. The velocity was zero at the outer wall of the NMR tube and maximum at the inner wall of the rotating bob, consistent with no-slip boundary conditions. The double slice selection reveals a velocity profile along a diameter that is antisymmetric. For clarity of discussion, the profile along one radius with a positive flow velocity is shown.

**Figure 3:**
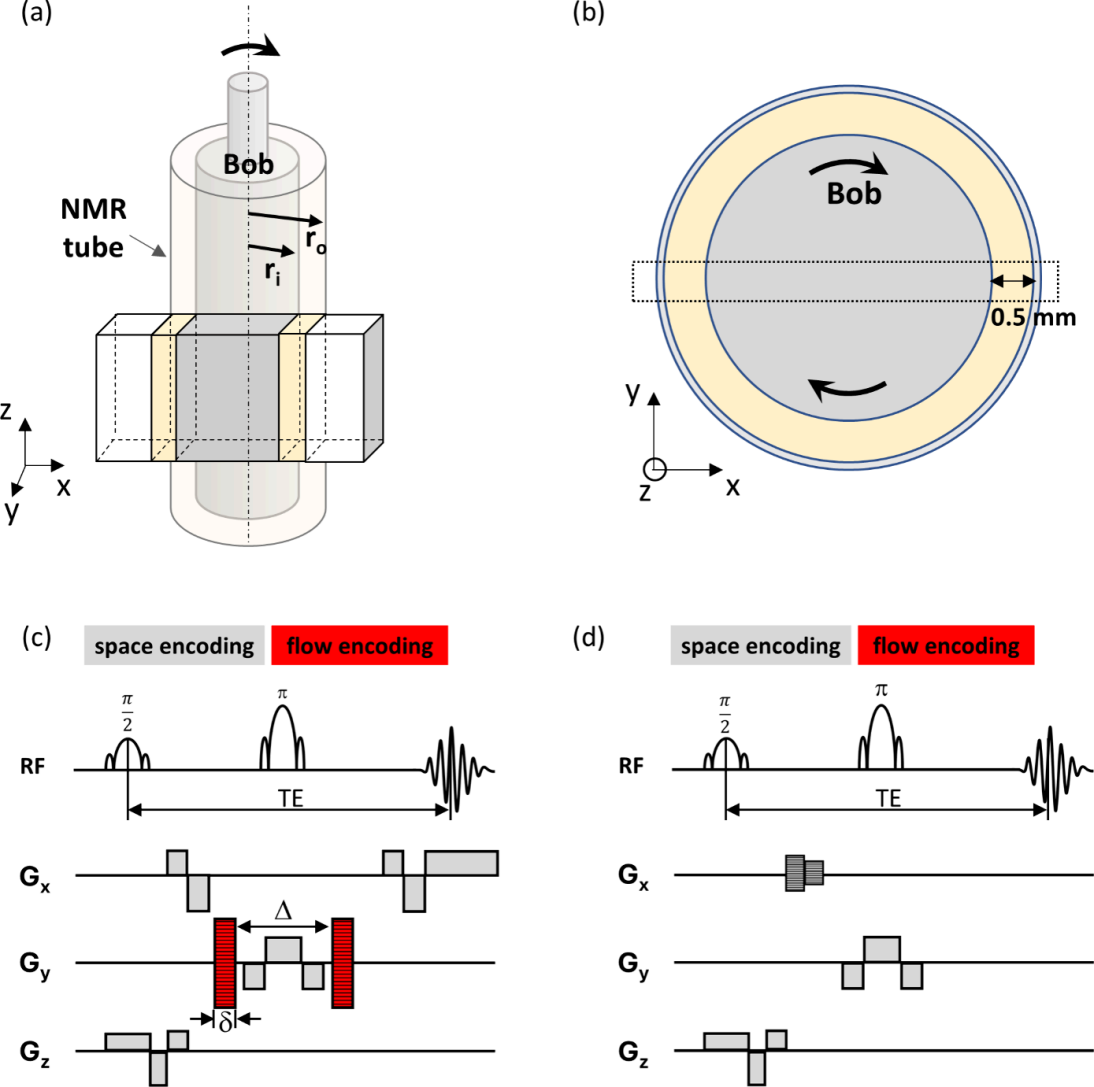
Schematic of the rheo-NMR and MRI velocimetry experimental setup. (a) Concentric double cylinders with inner and outer cylinder radii of *r*_i_ = 3.9 mm and *r*_0_ = 4.4 mm, respectively. The inner cylinder (bob) was rotated, while the outer cylinder was stationary. A 12 mm thick slice was selected along the vertical direction (*z*-axis) and a 1 mm thick slice was selected in the velocity direction (*y*-axis) to acquire data along the *x*-direction with a field of view of 12 mm in the imaging region (yellow-shaded box). (b) Top view of a cross section of the cell, showing the excitation volume and the gap of 0.5 mm (dashed box). NMR pulse sequences for one-dimensional space encoding using double-slice selection were used to measure (c) tangential ^1^H velocity profiles and (d) ^1^H oil concentration profiles of PCM nanoemulsions. The velocity and oil concentration profiles were acquired using pulsed gradient spin–echo (PGSE) sequences, which excited a 12 mm × 1 mm intersection between two orthogonal slices. To correct for flow-related dephasing, flow compensation gradients were incorporated. RF indicates the radio frequency, TE is the echo time in the spin–echo experiment, and G_*x*_, G_*y*_ and G_*z*_ are the three orthogonal magnetic field gradients.

To facilitate the comparison between two different frequencies, we computed normalized velocity profiles by dividing the velocities at each point by the maximum velocity near the inner wall (Figure 4), yielding normalized velocities between zero (outer wall) and one (inner wall). Velocity profiles of PCM nanoemulsions measured with a shear rate of 14 s^–1^ show a flow profile that is linear in the radial direction at 40, 26, and 17 °C upon cooling (Figure 4a). Under these conditions, we observed that the PCM nanoemulsions exhibited homogeneous and Newtonian fluid-like behavior, similar to the normalized velocity profile of pure water (Figure S2). After cooling and subsequent heating, at 26 °C, two distinct flow regions became evident, where a nonuniform velocity profile was observed within the gap. At this point in the temperature cycle, the liquid fraction of octadecane was 0.20. This transition to a non-Newtonian flow regime, characterized by the separation of the fluid into regions with varying shear rates,^31^ reveals underlying hydrodynamic instabilities. As the liquid fraction of octadecane increases while heating, this phenomenon diminishes at 27 °C, while the fluid velocity profile returns to near-linear behavior at 40 °C when the octadecane becomes fully melted.

**Figure 4:**
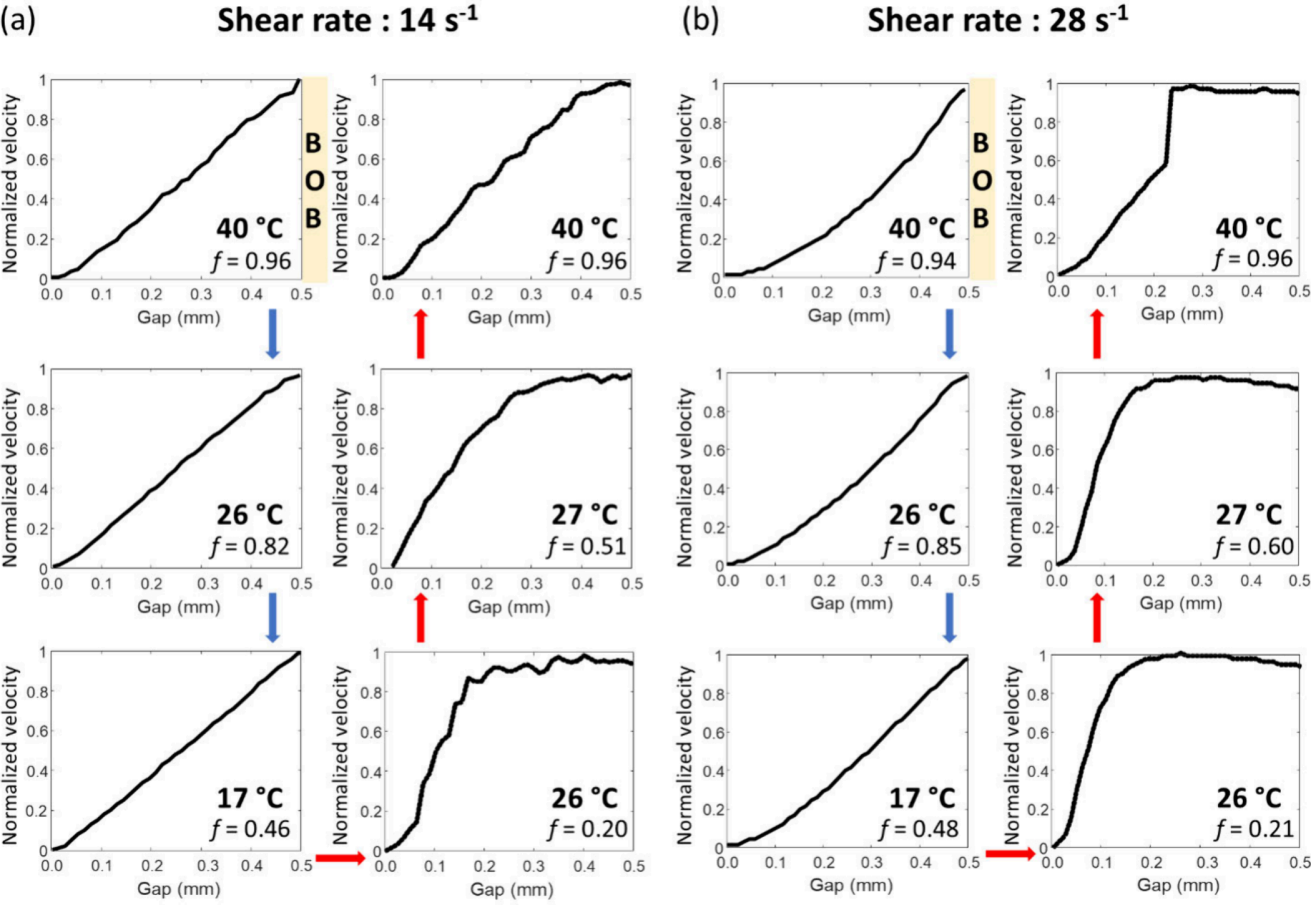
Normalized ^1^H velocity profiles of PCM nanoemulsions measured across the gap during thermal cycling using (a) a shear rate of 14 s^–1^ (bob rotation frequency of 0.25 Hz) or (b) a shear rate of 28 s^–1^ (bob rotation frequency of 0.50 Hz). Samples were cooled from 40 to 5 °C, then subsequently heated to 40 °C, under constant rotation. The yellow box on the right-hand side of the velocity profile indicates the position of the rotating bob. The temperature and liquid fraction of octadecane (*f*) are labeled in each velocity profile.

Interestingly, the velocity profiles measured using the same experimental procedure, but at twice the shear rate of 28 s^–1^ (Figure 4b), exhibited a modest deviation from linear behavior (convex) during cooling at temperatures of 40, 26, and 17 °C. During the cooling process and upon subsequent heating, the nonuniform fluid velocity profile once again appeared at 26 °C, when the liquid fraction of the octadecane was 0.21. However, unlike the results observed at a shear rate of 14 s^–1^, the nonlinear fluid velocity profile remained unchanged at 27 °C. Strikingly, upon heating to 40 °C, the non-Newtonian behavior became more pronounced instead of returning to its original flow profile, completely losing memory of its original flow state. Clearly, the combined effects of shear and temperature cycling have a significant effect on flow instabilities in the PCM nanoemulsions.

The velocity profiles that deviate from linearity can be divided into two regions with different slopes. In the first region near the rotating bob, the velocity gradients are small, resulting in a lower average local shear rate. In this relatively flat region of the velocity profile, the local viscosity of the fluid is higher than the average viscosity of the PCM nanoemulsion measured by the rheometer.^29^ In the second region close to the static wall, the velocity profile is linear and decreases to zero at the outer wall, leading to an average viscosity lower than that in the flat region. The large changes in local velocity suggest that unstable flow occurred due to shear-induced mass transport within the gap.

During thermal cycling, the effects of shear on the PCM nanoemulsion are more significant below the melting temperature of octadecane, consistent with the larger changes in droplet size observed at 25 °C vs 40 °C (Figure 2b). This result implies that shear-induced flow instability highly depends on the presence of solid octadecane, specifically the coexistence of solid and liquid octadecane, and thus the temperature and thermal history of the material. As octadecane melts, the velocity profile returns to its original state of flow at a shear rate of 14 s^–1^ but does not return at the higher shear rate of 28 s^–1^. This observation indicates that the effect of shear on PCM nanoemulsions becomes more pronounced at greater share rates.

In micellar solutions, nonuniform fluid velocity profiles (e.g., shear banding) have been understood in terms of shear-induced changes in microstructure,^29^ ordering of molecules,^21^ or aggregation of molecules,^31^ leading to localized changes in molecular viscosity in wormlike micellar solutions. For PCM nanoemulsions, shear-induced microstructural changes coupled with thermodynamic phase changes result in a complex flow behavior. To understand the origins of the non-Newtonian behavior and nonlinear velocity profiles observed during thermal cycling under shear, we measured the concentration distribution of octadecane within the gap immediately following the velocity profile measurements. Spatially resolved ^1^H NMR spectra were acquired using the pulse sequence in Figure 3d during thermal cycling for both shear rates (spectra shown in Figure S3). The integrated ^1^H signal intensities of the alkyl signals relative to the H_2_O signal were obtained within the gap and plotted relative to the maximum for each temperature to depict the concentration distribution of oil in the gap (Figure 5).

**Figure 5:**
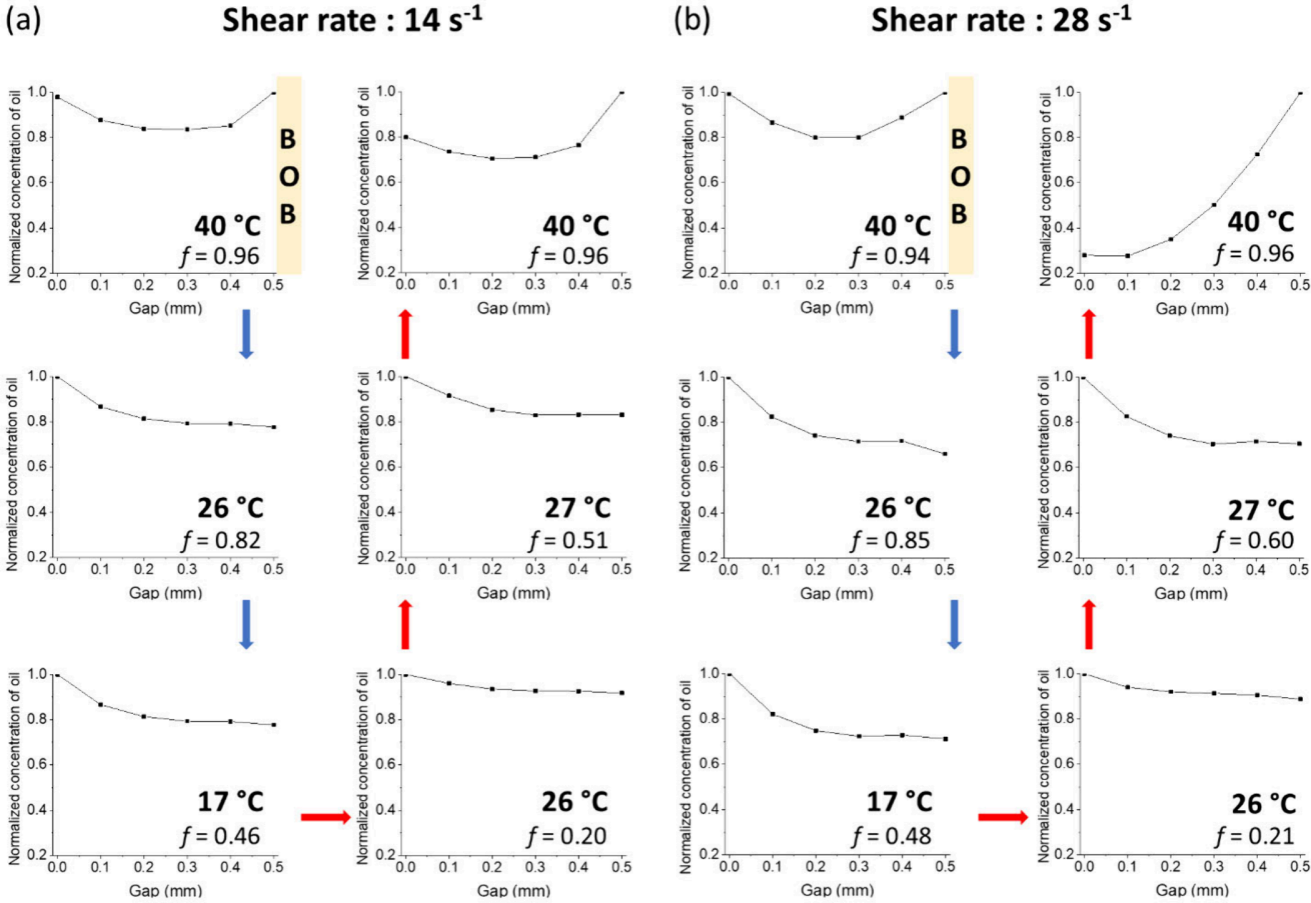
Normalized concentration distribution of liquid octadecane in PCM nanoemulsions measured across the gap during thermal cycling using (a) a shear rate of 14 s^–1^ (bob rotation frequency of 0.25 Hz) or (b) a shear rate of 28 s^–1^ (bob rotation frequency of 0.50 Hz). The yellow box on the right-hand side of the velocity profile indicates the position of the rotating bob. The temperature and liquid fraction of octadecane (*f*) are labeled in each concentration distribution.

The liquid oil concentration distribution is symmetrical across the gap at 40 °C at a shear rate of 14 s^–1^ (Figure 5a). Interestingly, the liquid oil concentration is the same near the static wall and the rotating bob, while it is modestly lower in the center region. While cooling, the oil concentration near the rotating bob decreases at 26 and 17 °C, indicating that the liquid oil droplets migrate toward the static wall. After cooling and subsequent heating at 26 °C, where the velocity profile became nonlinear, the liquid oil concentration (*f* = 0.20) near the rotating bob increases, resulting in a uniform concentration distribution within the gap. This result suggests a change in the direction of migration of liquid oil droplets toward the rotating bob. The liquid oil concentration near the bob slightly decreases at 27 °C and increases at 40 °C, showing local fluctuations in the concentration during the heating process.

A similar trend in the concentration distribution of liquid oil was measured at a shear rate of 28 s^–1^ (Figure 5b) compared to that at a shear rate of 14 s^–1^, except for the result obtained at 40 °C after a complete thermal cycle. Under these conditions, the oil concentration distribution near the static wall and near the bob exhibits significant differences. The PCM nanoemulsion phase separated into an oil-rich region near the rotating bob and a water-rich region near the static wall. This result indicates a highly nonuniform oil concentration profile, which would in turn significantly affect the local viscosity and therefore the velocity profile (Figure 4b). This behavior aligns with our observation of phase separation in the PCM nanoemulsion and the presence of aggregates on the bob after thermal cycling at a shear rate of 28 s^–1^ (Figure S4). Note that, at both rotation frequencies, nonlinear velocity profiles were observed at 26 and 27 °C upon heating, where the liquid oil concentration at the bob fluctuated between 26 and 27 °C. Overall, shear-induced mass transport occurred within the gap during thermal cycling, resulting in viscosity gradients that caused non-Newtonian behavior and nonlinear fluid velocity profiles.

In conclusion, rheo-NMR and MRI velocimetry, in conjunction with viscosity and dynamic light scattering measurements, have been applied to investigate the simultaneous effects of thermal cycling and shear on a model PCM nanoemulsion consisting of octadecane, water, and stearic acid. The PCM nanoemulsions were shear-thinning, exhibiting lower viscosities at higher shear rates. The mean droplet size increased after shear at 25 °C where liquid and solid octadecane coexist, a sign of phase instability, but not at 40 °C, when only liquid octadecane is present. The viscosity of the PCM nanoemulsion exhibited hysteresis during thermal cycling, which was correlated to the solid fraction of octadecane present within the nanoemulsion droplets.

Nonlinear velocity profiles developed upon the octadecane solid-to-liquid phase transition, which recovered to linear profiles upon complete octadecane melting at lower shear rates but not at higher shear rates. Concentration distributions of liquid octadecane obtained from spatially resolved liquid-state ^1^H single-pulse NMR spectra revealed nonuniform oil concentrations, indicating that shear-induced mass transport occurred during thermal cycling. The nonuniform octadecane concentration distributions give rise to gradients in local viscosity, leading to non-Newtonian behavior and the emergence of nonlinear fluid velocity profiles. Overall, this study highlights how shear affects flow instabilities in PCM nanoemulsions during thermal cycling and provides valuable insights for optimizing PCM nanoemulsions and operating conditions to guide their development as thermal energy storage systems. In addition, rheo-NMR and MRI velocimetry methods are shown to be powerful tools for noninvasively measuring flow and concentration profiles in complex fluids, including those that undergo thermodynamic phase transitions such as PCM nanoemulsions.

## Methods

### Materials

Octadecane (99%, melting temperature *T*_m_ = 26–29 °C), stearic acid (98.5%, *T*_m_ = 67–72 °C), deuterium oxide (99.9%), and anhydrous sodium hydroxide (NaOH) were purchased from the Millipore Sigma. Deionized water (DIW) was used (18 MΩ Millipore).

### PCM Nanoemulsions Formulation

The PCM nanoemulsion was synthesized by the following procedure. First, octadecane was premelted in an oven at 40 °C. The heating plate temperature (IKA RET basic C) and the ultrasonic bath temperature (Bandelin sonorex, 35 kHz) were both set to 75 and 70 °C, respectively. Stearic acid was weighed onto a glass vial, and then molten octadecane was introduced into the vial using a micropipette on the heated plate. The resulting mixture was then transferred to an ultrasonic bath and maintained at 70 °C for 5 min. As soon as the stearic acid was completely dissolved in octadecane, the solution became transparent. A solution of aqueous 0.05 M NaOH was prepared and placed on the heating plate, where it was maintained at 75 °C. Once the solution reached the desired temperature, it was carefully added to the octadecane and stearic acid mixture using a pipet. This mixture was sonicated in an ultrasonic bath for 60 min at 70 °C. Following sonication, the emulsion was cooled in a water bath at ambient temperature and then stored.

### Measurement of Average Droplet Size

The average droplet size and distribution were measured by dynamic light scattering (DLS) using a Malvern Nano ZS instrument as an indicator of the emulsion stability. The measurement was carried out at a scattering angle of 173° in polystyrene disposable cuvettes. PCM nanoemulsions were diluted 0.1% by volume in the DIW and the measurements were conducted after following a 120 s temperature equilibration time with a refractive index of 1.439 at 25 °C. For all experiments, we used freshly prepared nanoemulsions showing a monodisperse distribution and have mean diameter sizes range from 170 to 200 nm measured by DLS technique as seen in Figure S5.

### Measurement of Apparent Viscosity

Before the viscosity measurement, the evaporation test was performed since the rheometer was an open system (Table S1). A rotational rheometer (ARES-G2, TA Instruments) was used to examine the viscosity of PCM nanoemulsions with cup and bob geometry, 30 and 27.7 mm in diameter, respectively. A PCM nanoemulsion containing 20 wt % octadecane was used, which shows better stability. The distance between the lower tip of the bob and the bottom of the cup was 5.917 mm. Freshly prepared PCM nanoemulsions were employed with a sample volume of 22 mL. The temperature was controlled to within ± 0.1 °C by a Peltier system, and the heating rate was 0.5 °C/min. The dynamic viscosity of PCM nanoemulsion was measured with shear rate from zero to 1400 s^–1^ at constant temperature 25 °C. The apparent viscosities of PCM nanoemulsions were examined at 25 and 40 °C with different fixed shear rates of 500 and 1400 s^–1^. To investigate the thermal-mechanical stability of the PCM nanoemulsion, the apparent viscosities were measured from a fixed shear rate (500 s^–1^) during thermal cycling. Samples were cooled from 40 to 10 °C then heated up to 40 °C to change the phase of octadecane from solid to liquid while maintaining water in the liquid phase.

### Rheo-NMR and MRI Velocimetry

Rheo-NMR and MRI velocimetry measurements were performed with a Bruker AVANCE I NMR spectrometer with a 7.05 T wide bore magnet operating at a Larmor frequency of 300.13 MHz for protons. A Bruker Micro 5 microimaging probe with a 10 mm saddle coil was used that generates pulsed magnetic field gradients of up to 2.0 T/m in three orthogonal dimensions. The PCM nanoemulsion samples (1 mL) were loaded into a 10 mm NMR tube with an inner diameter of 8.8 mm. Then, a 7.7 mm polyether ether ketone (PEEK) bob was inserted coaxially into the NMR tube. The schematic image of the experimental set up is depicted in Figure 3, illustrating (a) the concentric double cylinder with inner and outer radii (*r*_i_ = 3.9 mm and *r*_o_ = 4.4 mm, respectively) and (b) a top view of a cross-section of the cell, showing the gap (0.5 mm). Shear was applied to the sample using an in-house developed rheo-NMR system similar to the one utilized by Kohn et al.,^32^ except that it omitted the gearbox for oscillatory motion. Rotation of the bob is generated by a servo motor, and the drive shaft connecting to the bob ensured vibration-free rotation. A ^1^H radiofrequency (rf) field strength of 25 kHz was used for all experiments, corresponding to a 90° pulse of 10 μs. A temperature equilibration time of 15 min was used, which was determined experimentally under constant shear conditions.

To measure ^1^H velocity profiles, a 12 mm × 1 mm selection column was excited by applying a double-slice selective pulse sequence using selective rf soft pulse as depicted in Figure 3c.^32^ The relatively thick slice in the vertical direction is possible because of the cylindrical symmetry, thereby allowing for better signal intensity. To correct for flow-related dephasing, we introduced additional flow compensation gradients into all of the space-encoding gradients. A selective three-lobed sinc-type rf pulse (π/2) was applied under a gradient in the *z*-direction, producing a slice with a thickness of 12 mm. A selective three-lobed sinc-type refocusing rf pulse (π) selected a slice thickness of 1 mm along the *y*-direction. Local velocities within the excited slice in the *y*-direction were measured along the *x*-direction with a field of view of 12 mm, and 1024 points were collected. This results in a spatial resolution of 12 μm per pixel with a spatial resolution of about 30 μm. The experiments were carried out using a total echo delay time of TE = 15 ms. The diffusion time was Δ = 3.3 ms between flow-encoding gradients, each of which had a duration of δ = 2 ms. The velocity encoding gradients were linearly incremented from −11.5 to 11.4 G mm^–1^ in 128 steps. The gradient pulses were trapezoidal and had a ramp time of 80 μs. A recycle delay of 2 s was used between scans, which was approximately two times the ^1^H longitudinal relaxation time (*T*_1_) of the PCM nanoemulsion proton signals to decrease the experiment time. Flow profiles were measured at different temperatures during thermal cycling with a 0.25 or 0.50 Hz rotation frequency of the bob, which resulted in shear rates of 14 or 28 s^–1^, respectively. The field of flow was chosen according to the rotation speed of the inner bob to accommodate more than twice the maximum tangential velocity of the rotating bob. The peak maximum in the velocity dimension was extracted as the velocity for the component measured in each experiment for every pixel.

^1^H spatially resolved NMR spectra were obtained by using the pulse sequence in Figure 3d under flow without velocity encoding to obtain the concentration distribution of oil within a gap. Two signals are resolved, water and the alkyl signal attributed to the oil and surfactant. The area under a specific signal in the spectrum is directly proportional to the quantity of spins with the corresponding molecular structure in the sample, providing information about the concentration of that component. The experiments were performed with a recycle delay of 8 s and echo time TE = 7 ms following the measurements of flow profile.

Liquid-state ^1^H single-pulse NMR spectra were obtained without water suppression using a recycle delay of 8 s to ensure the complete relaxation of all nuclear spins between pulses, which was more than five times the ^1^H longitudinal relaxation time, *T*_1_, of the PCM nanoemulsion proton signals.^33^ The relative intensity of the total ^1^H integrated area of the alkyl signal, divided by that of the water signal, was calculated to obtain the liquid fraction of octadecane at different temperatures.

Data processing was performed using TopSpin and in-house written MATLAB scripts that included functions from the free software package matNMR.^34^

## Data Availability

Data are available upon request.

## Supporting Information Available

Dynamic viscosity measurements, normalized velocity profiles of water, spatially resolved ^1^H NMR spectra during thermal cycling at different shear rates, images of cell after rheo-NMR measurements, DLS measurements of emulsion size distributions, evaporation test results (PDF)
