# Supplementary material for: Simultaneous Effects of Thermal Cycling and Shear on Flow Instabilities of Phase-Change Nanoemulsions Measured by Rheo-NMR and MRI Velocimetry
Source: J Phys Chem Lett. 2025 Mar 24;16(13):3316–25. doi: 10.1021/acs.jpclett.5c00307 (PMC11973913; doi:10.1021/acs.jpclett.5c00307)
Supplement: Supplementary file 1 — jz5c00307_si_001.pdf [file jz5c00307_si_001.pdf]

Supporting Information

Simultaneous Effects of Thermal Cycling and Shear on Flow Instabilities of Phase-Change Nanoemulsions Measured by Rheo-NMR and MRI Velocimetry

Jungeun Park[1], Benjamin Kohn[2,†], Robert J. Messinger[1,*], Ulrich Scheler[2,*]

[1]*Department of Chemical Engineering, The City College of New York, CUNY, New York, NY, 10031, USA*
[2]*Department for Multi-Scale Characterization, Leibniz-Institut für Polymerforschung Dresden e.V., Hohe str. 6, 01069, Dresden, Germany*
[†]*Present Address: Department of Radiology, Washington University in Saint Louis, St. Louis, Mo 63110, USA*
*Email: rmessinger@ccny.cuny.edu, scheler@ipfdd.de

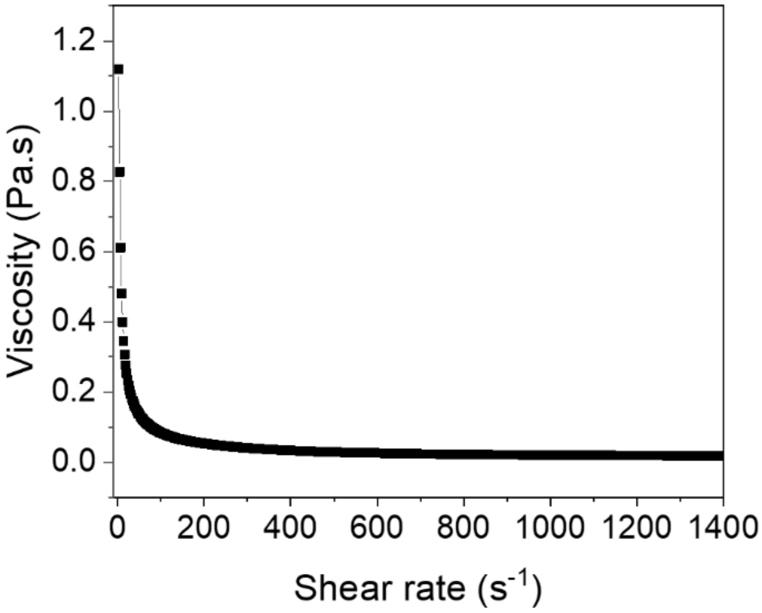

Figure S1. Dynamic viscosity of PCM nanoemulsion composed of 20 wt. % octadecane, 2.5 wt. % stearic acid, 77.5 wt. % aqueous 0.05 M NaOH as a function of shear rate measured at 25 °C.

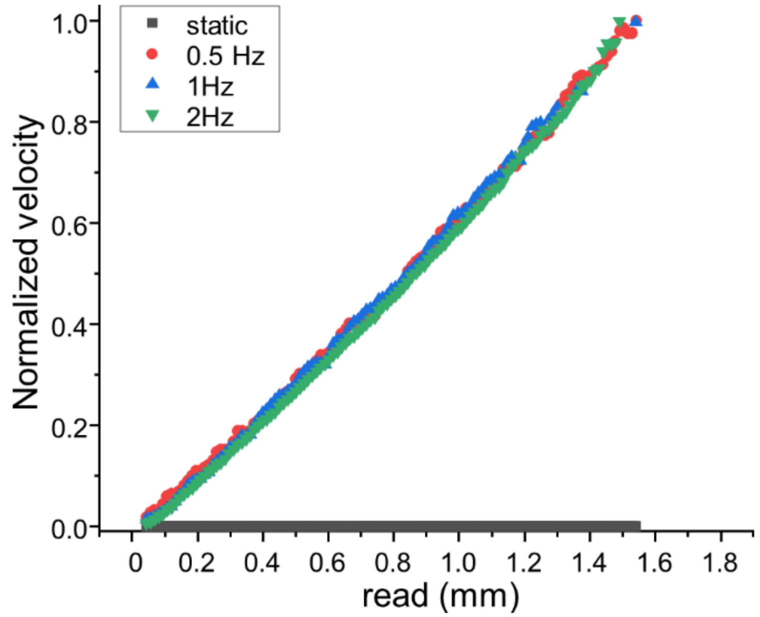

Figure S2. Normalized velocity profiles of water across the 1.8-mm gap of a 5-mm/ 8.8-mm diameter concentric double cylinder with rotation frequency of 0.0, 0.5, 1.0, and 2.0 Hz at room temperature. A small quantity of $CuSO_4$ (1 g/L) was added to shorten the ^1H longitudinal (T_1) NMR relaxation time of the water to facilitate a shorter recycle delay (0.5 s) between scans.

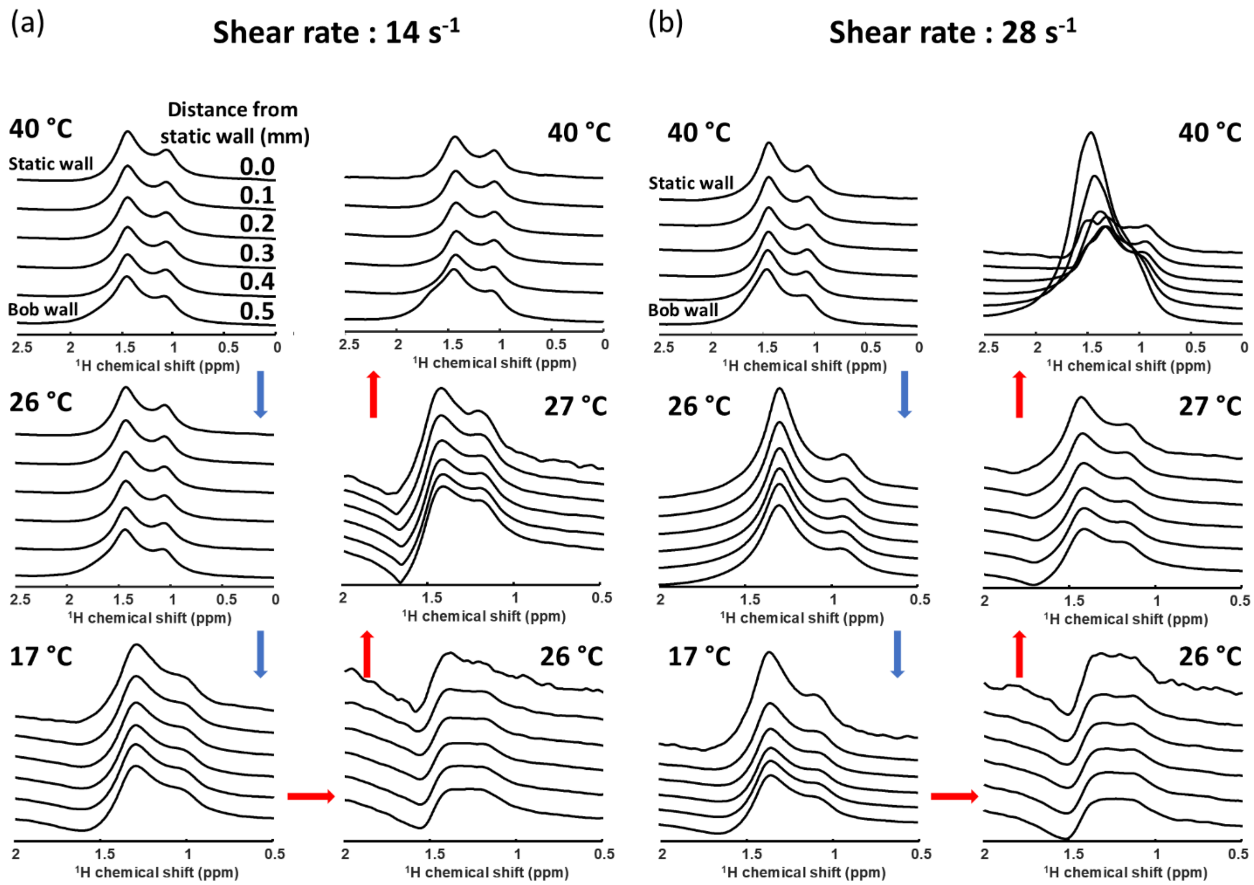

Figure S3. Spatially resolved ^{1}H NMR spectra of octadecane in the PCM nanoemulsion across the gap of the cell acquired using rotation rates of (a) 14 s^{-1} and (b) 28 s^{-1} during thermal cycling. ^{1}H chemical shifts were referenced to the H_2O signal at 4.8 ppm.

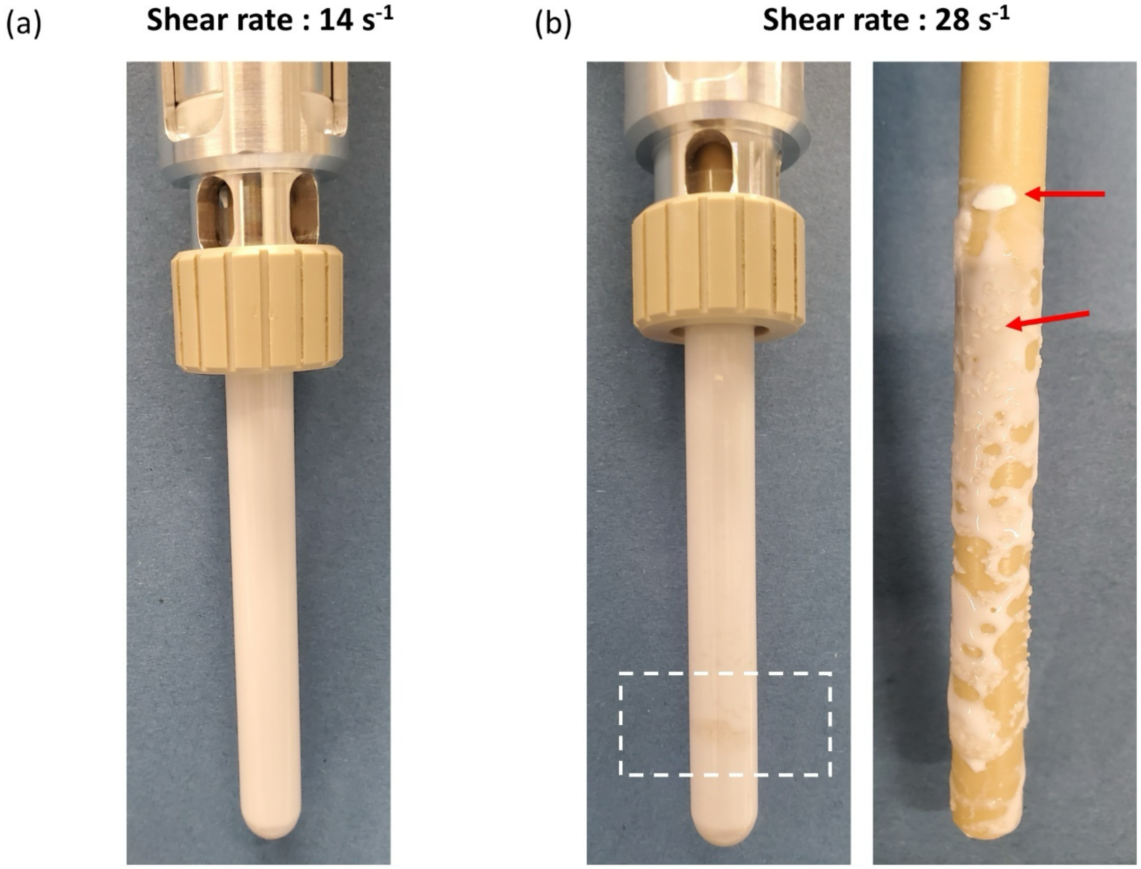

Figure S4. Pictures of the cell after the rheo-NMR measurements using different rotation rates: (a) 14 s^{-1} and (b) 28 s^{-1}. After thermal cycling at 14 s^{-1} phase separation was observed within the cell as well as the presence of aggregates of the PCM nanoemulsion on the bob (inner cylinder).

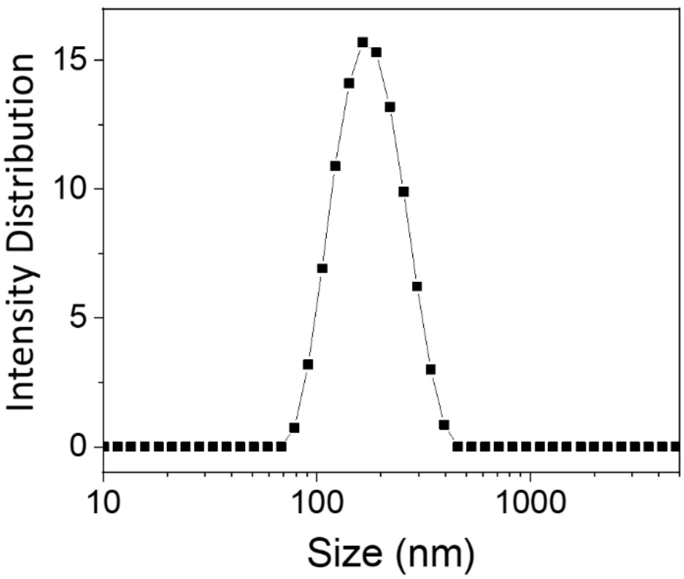

Figure S5. Droplet size distribution measured by dynamic light scattering (DLS) of a PCM nanoemulsion composed of 30 wt. % octadecane, 3.75 wt. % stearic acid, 66.25 wt. % aqueous 0.05 M NaOH used for concentration and flow measurements. The refractive index was 1.439 at 25 °C. The mean diameter size was 170 nm and polydispersity index (PDI) was 0.142, indicating a monodisperse distribution.

Table S1. Calculated weight loss of octadecane, deionized water (DIW), and PCM nanoemulsion from an evaporation test. The exposed area of the vial and the rheometer were 176.7 and 104.2 mm^2, respectively.

**Vial at 40 °C**		**Octadecane (wt %)**	**DIW (wt %)**	**Emulsion (wt %)**
Mass loss	1 h	0.006	0.16	0.14
	17 h	0.007	3.00	1.86
**Rheometer at 40 °C**		**Octadecane (wt %)**	**DIW (wt %)**	**Emulsion (wt %)**
Mass loss	1 h	0.004	0.10	0.08
	17 h	0.004	1.77	1.10
